# The experience of felt presence in a general population sample

**DOI:** 10.1192/bjp.2024.7

**Published:** 2024-04

**Authors:** Sanne G. Brederoo, Ben Alderson-Day, Janna N. de Boer, Mascha M. J. Linszen, Iris E. C. Sommer

**Affiliations:** University Center of Psychiatry, University Medical Center Groningen, University of Groningen, The Netherlands; Department of Psychology, Durham University, UK

**Keywords:** Felt presence, hallucinations, psychosis, risk factors, general population

## Abstract

Felt presence is a widely occurring experience, but remains under-recognised in clinical and research practice. To contribute to a wider recognition of the phenomenon, we aimed to assess the presentation of felt presence in a large population sample (*n* = 10 447) and explore its relation to key risk factors for psychosis. In our sample 1.6% reported experiencing felt presence in the past month. Felt presence was associated with visual and tactile hallucinations and delusion-like thinking; it was also associated with past occurrence of adverse events, loneliness and poor sleep. The occurrence of felt presence may function as a marker for general hallucination proneness.

Felt presence (also referred to as ‘sensed presence’) is the experience of an external entity in the nearby environment without clear sensory evidence.^1^ Although typically associated with Parkinson's disease,^2^ felt presence is increasingly recognised as a transdiagnostic phenomenon occurring in schizophrenia-spectrum disorders, neurological diseases and under psychologically or physically taxing conditions.^1^ Felt presence is closely linked to body-related hallucinatory phenomena^3^ and to ultra-high risk for psychosis.^4^

Despite growing awareness, felt presence continues to be under-recognised in clinical and research contexts. It is poorly understood compared with more widely known psychotic experiences, and is sometimes described as falling between hallucinations and delusions.^5^ Currently, reports of felt presence are limited to case studies (summarised in Barnby et al^1^) and small-scale phenomenological studies.^6^ So far, no studies have examined felt presence in a large population sample and little is known about its wider non-clinical presentation.

A few years ago, such data were gathered as part of a study by Linszen et al^7^ in which psychotic-like experiences were assessed via self-report in a large sample from the general Dutch population. Given previously noted associations with the development of psychosis, we hypothesised that felt presence should be understood as a hallucination-like experience and that it would show similar associations with key risk factors for hallucinations. Therefore, in the current study, we tested for the expected relationship of felt presence with other forms of hallucinations and delusions in this sample, and for associations with key psychosis risks, including adverse events, loneliness and sleep disruption.

## Method

In a national online survey among the general Dutch population,^7^ 10 447 participants aged ≥14 years rated their propensity for psychotic-like experiences (69% female; median age 32 years, range 14–88 years) using the Questionnaire for Psychotic Experiences. Of these, 165 (1.6%) reported experiencing felt presence in the past month in response to a question on the presence of hallucination-like experiences (Appendix) and a follow-up free-text question asking participants to describe such experiences. A content analysis of the free-text responses was performed to derive codes further categorising the replies. In addition, we asked about the number of years since their first felt presence, past adverse events (not further specified) and experiences of other hallucinations (auditory, visual, tactile, and olfactory) and delusion-like thinking. Furthermore, we compared people who experienced felt presence with a subgroup of the larger sample who reported hallucination-like experiences in the past month but did not report felt presences (*n* = 5169) (‘noFP’ group). We explored associations with loneliness and sleep using the De Jong Gierveld Loneliness Scale^8^ and Pittsburgh Sleep Quality Inventory.^9^

The authors assert that all procedures contributing to this work comply with the ethical standards of the relevant national and institutional committees on human experimentation and with the Helsinki Declaration of 1975, as revised in 2008. The medical ethical committee of the University of Utrecht (IRB number 16–408/C) exempted the study from medical ethical review owing to its non-invasive and non-medical nature. All participants gave written informed consent before participation.

## Results

Of the 165 participants who had experienced felt presence in the past month, 85% were women. This proportion is significantly larger than in the group with hallucination-like experiences but no felt presence – the noFP group (75%) (*χ*^2^ = 7.65, *P* = 0.006). There was an over-representation of felt presence among those aged under 30, but not above that age (*t* = 2.77, *P* = 0.006). The median age of the participants who had experienced felt presence was 26 years (IQR = 19) and median age at which the first experience of felt presence occurred was 12 years (IQR = 10).

Most participants (72%) experienced felt presence not more than once a month; 22% reported weekly felt presence. Few participants reported daily (4%) or continuous felt presence (2%). A quarter of participants indicated that their felt presence had some relation with a past adverse advent, whereas only 5% of the noFP group reported such a relation for other hallucination-like experiences (*χ*^2^ = 116, *P* < 0.001). No further details about these adverse events were collected.

**Fig. 1:**
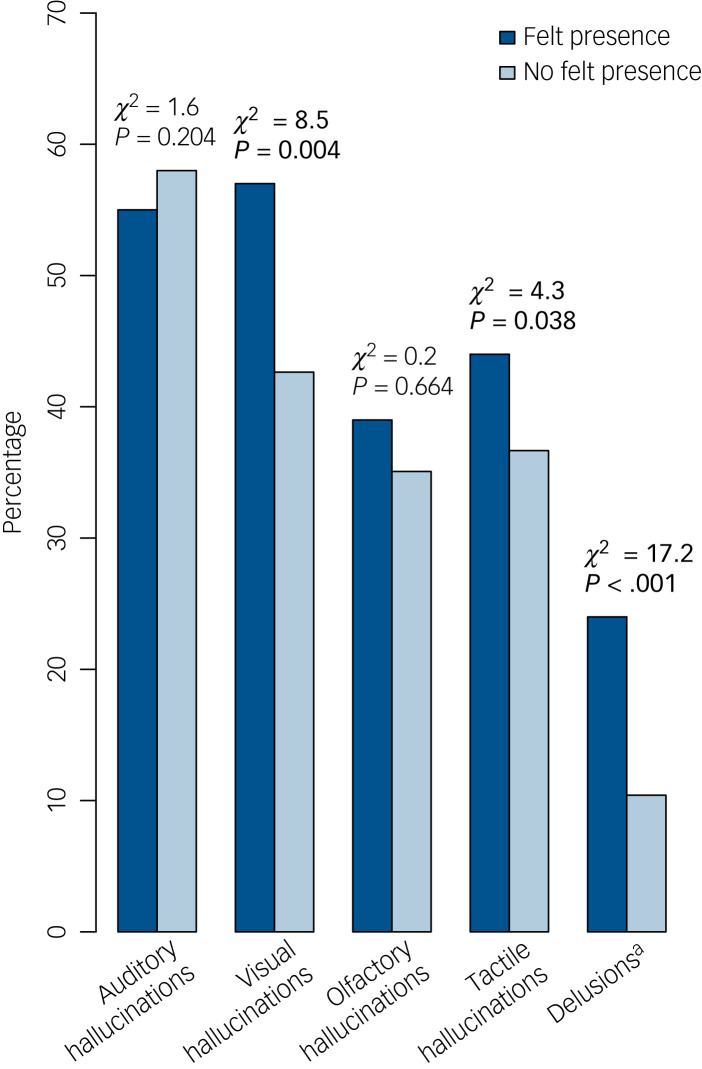
Percentages of participants who reported at least one hallucination-like experience in a particular sensory modality or delusional thinking in the past month. Felt presence is not counted among the listed experiences: instead, the participants are separated into those who experienced felt presence (FP) in the past month (*n* = 165) and those who did not (noFP) (*n* = 5169). Statistics from *χ*^2^-tests comparing the felt presence and noFP groups are given, with significant group differences in bold. a. A subset of participants filled out questions regarding the presence of delusions: *n* = 100 individuals (61%) in the FP group and *n* = 3276 (63%) in the noFP group.

When compared with the noFP group, people who experienced felt presence showed a higher prevalence of additional visual and tactile hallucinations in the past month, as well as delusion-like thinking (specifically delusions of reference) (all *χ*^2^ > 4.3, all *P* < 0.039) (Fig. 1). The prevalence of auditory and olfactory hallucinations did not differ between the felt presence and noFP groups (all *χ*^2^ < 1.61, all *P* > 0.203). Compared with the noFP group, experiencing felt presence in the past month was associated with greater loneliness (*t* = 1.92, *P* = 0.030) and poorer sleep quality (*t* = 2.74, *P* = 0.007).

When asked to describe their felt presences, participants typically reported ‘sensing’ that someone was there without seeing anyone. In some cases, participants indicated that they felt as if they were being watched (18%) or they concurrently felt a hand on their shoulder (12%).

## Discussion

Our main objective was to offer a view of felt presence as it occurs in a general population sample. As hypothesised, we found that individuals who experienced felt presence also experienced other types of hallucination, along with delusional ideas. Well-known risk factors for hallucinations – adverse events, loneliness and poor sleep quality – were also more prevalent among people with felt presence. These findings corroborate the theory that felt presence shares a common underlying mechanism with other psychosis-like experiences. Felt presence might therefore be seen as a marker for general hallucination proneness.

Concurrent reports of overlap with other senses, such as feeling a hand on a shoulder, can be hypothesised to strengthen the experience of the presence of an ‘other’ entity, in which the felt presence is focused on the individual. Rather than sensing a passively present entity, the experiencer feels as if they are at the centre of the presence's attention, as evidenced by accounts of ‘being watched’ or touched by the felt presence. This tallies with our finding of a specifically high rate of delusions of reference among people who experience felt presence.

Although the significant association of felt presence with visual and tactile but not auditory hallucinations suggests a profile more typical of hallucinations in Parkinson's disease,^2^ this is difficult to reconcile with the relatively young age of the participants. Previously, Larøi et al^10^ also reported the lack of an association between felt presence and auditory hallucinations, and found felt presence to be more common in those also experiencing tactile and olfactory hallucinations, across the lifespan. Further research is needed to gain a better understanding of this weaker link between felt presence and hallucinations in the auditory domain compared with those in other senses.

### Limitations

It must be noted that response bias probably affects the current data-set, as participation in the survey might have been more appealing to people with psychotic experiences, leading to over-representation of these phenomena. In addition, women and younger people generally participate more often in online surveys, which was the case for the current study and resulted in a skewed sample.

### Further research and clinical implications

Although women were over-represented in this sample, our data confirm the previous observation that women may be more susceptible to experiencing felt presence than men.^11^ Future studies are needed to replicate and assess what drives this link, for example by examining gender differences in prevalence of certain types of adverse event.

By providing an account of felt presence as occurring in the general population, we aimed to contribute to its wider recognition among at-risk populations and clinical groups. As these are the experiences that patients say clinicians are currently missing,^12^ increasing awareness of the occurrence of felt presence is crucial going forward.

## Data Availability

The data that support the findings of this study are available from the corresponding author, S.G.B., on reasonable request. They are not made publicly available as they contain information that could potentially compromise participants’ privacy. The analytic code can also be requested with the data. There are no other materials supporting the findings that are available to other researchers.

## Appendix

Participants were asked about their experiences of hallucination-like phenomena in the tactile sensory domain: ‘Some people have experienced being touched by someone or feeling a hand on their shoulder while nobody was nearby. Another example is people having the feeling as if bugs are crawling under their skin. Have you ever experienced something like that? If so, have you experienced this in the past week? If not in the past week, have you experienced this in the past month?’. Of the 165 reports of felt presence most (89%) were in response to this question, but a small proportion of the felt-presence reports – or elaborations on felt presence – were given in response to similar questions about hallucination-like experiences in the visual (13%) and auditory (1%)^10^ domains.

## References

[1] Barnby JM, Park S, Baxter T, Rosen C, Brugger P, Alderson-Day B. The felt-presence experience: from cognition to clinic. Lancet Psychiatry 2023; 10: 352–62. 36990104 10.1016/S2215-0366(23)00034-2

[2] Fénelon G, Soulas T, Cleret de Langavant L, Trinkler I, Bachoud-Lévi AC. Feeling of presence in Parkinson's disease. J Neurol Neurosurg Psychiatry 2011; 82: 1219–24. 21551471 10.1136/jnnp.2010.234799 PMC3382202

[3] Rosen C, Park S, Baxter T, Tufano M, Giersch A. Sensed presence, attenuated psychosis, and transliminality: at the threshold of consciousness. Psychopathology 2023; 56: 359–70. 36754040 10.1159/000528572 PMC10534996

[4] Park S, Baxter T. Schizophrenia in the flesh: revisiting schizophrenia as a disorder of the bodily self. Schizophr Res 2022; 242: 113–7. 34996674 10.1016/j.schres.2021.12.031

[5] Koehler K, Sauer H. Jasper's sense of presence in the light of Huber's basic symptoms and DSM-III. Compr Psychiatry 1984; 25: 183–91. 6705509 10.1016/0010-440x(84)90007-5

[6] Alderson-Day B, Woods A, Moseley P, Common S, Deamer F, Dodgson G, et al. Voice-hearing and personification: characterizing social qualities of auditory verbal hallucinations in early psychosis. Schizophr Bull 2021; 47: 228–36. 33484268 10.1093/schbul/sbaa095 PMC7824995

[7] Linszen MMJ, de Boer JN, Schutte MJL, Begemann MJH, de Vries J, Koops S, et al. Occurrence and phenomenology of hallucinations in the general population: a large online survey. Schizophrenia 2022; 8: 41. 35853871 10.1038/s41537-022-00229-9 PMC9261095

[8] De Jong Gierveld J, Van Tilburg T. A 6-item scale for overall, emotional, and social loneliness. Res Aging 2006; 28: 582–98.

[9] Buysse DJ, Reynolds CF, Monk TH, Berman SR, Kupfer DJ. The Pittsburgh sleep quality index: a new instrument for psychiatric practice and research. Psychiatry Res 1989; 28: 193–213. 2748771 10.1016/0165-1781(89)90047-4

[10] Larøi F, Bless JJ, Laloyaux J, Kråkvik B, Vedul-Kjelsås E, Kalhovde AM, et al. An epidemiological study on the prevalence of hallucinations in a general-population sample: effects of age and sensory modality. Psychiatry Res 2019; 272: 707–14. 30832190 10.1016/j.psychres.2019.01.003

[11] Alderson-Day B, Moseley P, Mitrenga K, Moffatt J, Lee R, Foxwell J, et al. Varieties of felt presence? Three surveys of presence phenomena and their relations to psychopathology. Psychol Med 2023; 53: 3692–700. 35227337 10.1017/S0033291722000344 PMC10277754

[12] Pagdon S, Jones N. Psychosis outside the box: a user-led project to amplify the diversity and richness of experiences described as psychosis. Psychiatr Serv 2023; 74: 760–3. 36475822 10.1176/appi.ps.20220488

